# Cerebral Infarction as the Primary Presentation of Acute Aortic Dissection

**DOI:** 10.31083/j.rcm2406164

**Published:** 2023-06-06

**Authors:** Li-Ping Zhou, Xiang-Min Li, Guo-Qing Huang, Fang-Jie Zhang

**Affiliations:** ^1^Department of Emergency Medicine, Xiangya Hospital, National Clinical Research Center for Geriatric Disorders (Xiangya Hospital), Central South University, 410008 Changsha, Hunan, China

**Keywords:** aortic dissection, cerebral infarction, missed diagnosis, initial manifestation, emergency

## Abstract

**Background:**

The aim of this study was to determine the clinical characteristics and outcome of patients with aortic dissection (AD) who present with an initial manifestation of cerebral infarction.

**Methods:**

We retrospectively analyzed patients who were diagnosed with AD and admitted to the emergency department from May 1, 2017 to May 1, 2022. Data was collected for variables including age, sex, clinical manifestation, past medical history, and laboratory test results.

**Results:**

Twenty-five patients (2.61%, 22 type A and 3 type B) showed cerebral infarction as the primary presentation for acute AD, while another 933 AD patients (471 type A and 462 type B) who presented with other symptoms served as the control group. Eighteen of the 25 patients (72%) were initially diagnosed with stroke, and the diagnosis of AD was missed. However, patients with a missed diagnosis of AD did not have significantly different mortality to those in whom AD was diagnosed (chi-square test, *p *> 0.9999). Patients with cerebral infarction as the first presentation had a higher incidence of type A AD than the control patients (*p* = 0.0002), while their mortality rate was also higher than the control group of AD patients (*p *< 0.0001). Furthermore, patients with cerebral infarction as the first presentation were more likely to have multiple organ dysfunction.

**Conclusions:**

AD with an initial presentation of cerebral infarction is a rare condition with high mortality. However, the initial failure to diagnose AD does not further increase patient mortality.

## 1. Introduction

Aortic dissection (AD) is defined as disruption of the medial layer provoked by intramural bleeding, resulting in separation of the aortic wall layers and subsequent formation of a true lumen and a false lumen, with or without communication [1]. Acute aortic dissection (AAD) is a life-threatening vascular disease with high morbidity and mortality rates. Type A AD has a mortality rate of 50% within the first 48 hours, if not operated on. Despite improvements in surgical and anesthetic techniques, perioperative mortality and neurological complications remain high [2]. However, the clinical manifestations of AD vary due to the different types, scope, and extent of the tear location, as well as the influence of various underlying diseases. AD may also be clinically silent in many cases. A broad range of symptoms may be related to AD, including acute chest or back pain, cough, shortness of breath, abdominal pain or discomfort, a feeling of fullness, stroke, transient ischemic attack, hoarseness, limb ischemia, and so on [1, 3]. Such varied symptoms can easily be missed and misdiagnosed. It has been reported that emergency medicine physicians miss an AD diagnosis in 38% of cases, and in the cases they do identify correctly, 25% remained undiagnosed for >24 hours [4, 5]. If AD is left untreated, the mortality rate increases by 1–2% per hour from the initial presentation, and may reach as high as 40–70% in the acute period of 2 weeks [6].

The aortic arch gives off three branches from right to left: the brachiocephalic trunk (subdivided into the right common carotid artery and the right subclavian artery), the left common carotid artery, and the left subclavian artery. The common carotid artery is divided into the internal carotid artery and the external carotid artery, with the internal carotid artery branching to the optic and brain. The branches of the subclavian artery include the vertebral artery, which enters the cranial fossa through the foramen magnum and then branches to the brain and spinal cord. AD lesions can therefore lead to tearing or occlusion of the brachiocephalic trunk, common carotid artery [7] and subclavian artery [8], resulting in cerebral malperfusion [9] and even cerebral ischemia [10, 11].

Several studies have examined surgical management and outcomes in patients with AD and cerebral malperfusion or cerebral ischemia [12, 13]. However, there have been few studies on the misdiagnosis of cerebral ischemia as the initial manifestation of AD. The objectives of the present study were to investigate the clinical characteristics and outcome of AD patients with an initial manifestation of cerebral infarction.

## 2. Methods

The study protocol was approved by the ethics committee of Xiangya Hospital, Central South University, Changsha, China (No. 202210219). The study protocol complied with the guidelines of the Declaration of Helsinki (1964) and its later amendments. The requirement for written informed consent was waived as the data used in the study was retrospective and anonymous. Patients who were diagnosed with AD and admitted to the emergency department of Xiangya Hospital from May 1, 2017 to May 1, 2022 were retrospectively analyzed.

The inclusion criteria for AD patients with cerebral infarction as the initial manifestation were: (1) the patient initially had acute cerebral infarction-related symptoms (syncope, disturbance of consciousness, coma, weakness or hemiplegia on one side limb, speech impairment, headache, etc.) and later experienced chest/back/abdominal pain; (2) computed tomography (CT) and/or magnetic resonance imaging (MRI) confirmed new cerebral infarction changes; (3) no cerebral infarction was found by CT and/or MRI, but one of the brachiocephalic trunk (or right common carotid artery and right subclavian artery), left common carotid artery, or left subclavian artery was torn or occluded; (4) computed tomography angiography (CTA) or MRI confirmed acute AD. The exclusion criteria for the study were: (1) no cerebral infarction-related symptoms; (2) patients who had not undergone head imaging; (3) the patient had cerebral infarction symptoms, but imaging tests confirmed that none of the brachiocephalic trunk, left common carotid artery and left subclavian artery had tears or occlusion; (4) acute cerebral infarction-related symptoms occurred later than the chest/back/abdominal pain; (5) acute cerebral infarction-related symptoms occurred during the patients’ hospitalization period. If the patient met the 1+2+4 or 1+3+4 criteria and had no exclusion criteria, they were included in the study and recognized as AD patients with cerebral infarction as the initial manifestation. The Stanford AD classification was used in this study, which divides AD into type A (involvement of the ascending aorta), and type B (no involvement of the ascending aorta) [2]. As per the 2014 ESC Guidelines on the diagnosis and treatment of aortic diseases [1], the time course for AD is divided into acute (<14 days), subacute (15–90 days), and chronic (>90 days) phases.

### 2.1 Data Collection

Data was collected for the variables of age, sex, clinical manifestation, past medical history, and laboratory test results. The latter included routine blood tests (white blood cells, red blood cells, hemoglobin platelets neutrophil and lymphocytes), liver function, kidney function, coagulation, myocardial enzymology, total bilirubin, triglycerides, total cholesterol, high-density lipoprotein, low density lipoprotein, and C-reactive protein. All laboratory tests were performed within the first hour of patient admission to the emergency department (ED). Mortality and survival data of patients diagnosed with AD in the ED or before surgery were also collected. The German Registry of Acute Aortic Dissection Type A score (GERAADA score) was calculated for each patient according to https://www.dgthg.de/de/GERAADA_Score#. CT and/or MRI imaging reports were collected, but no data on the post-surgery survival of patients.

### 2.2 Statistical Analysis

Statistical data were analyzed using Prism 9 (GraphPad, San Diego, CA, USA) software. Data with normal distribution was represented as the mean ± standard deviation, with the Student’s *t*-test used to compare groups. The median was used for data with non-normal distribution [*M *(*Q*1, *Q*3)], and the Wilcoxon rank sum test was used to compare these groups. The comparison of count data between two groups was performed using Pearson’s chi-square test, with Fisher’s exact probability test used when appropriate. For all analyses, *p <* 0.05 was considered to be statistically significant.

## 3. Results

A total of 978 patients were diagnosed with AD in our ED from May 1, 2017 to May 1, 2022. Twenty cases were excluded because the AD type was not recorded or because no new dissection was found at the time of admission, even though the patient had previous dissection surgery. Finally, 958 patients were included in the study (493 type A and 465 type B). Of these, 25 patients (15 males and 10 females) met the inclusion criteria for cerebral infarction as the primary manifestation (22 type A and 3 type B), with the remaining 933 patients serving as the control group (471 type A and 462 type B). Therefore, 2.61% of the AD patients had cerebral infarction as the primary presentation, comprising 4.67% of the type A patients and 0.65% of the type B patients.

**Fig. 1.**
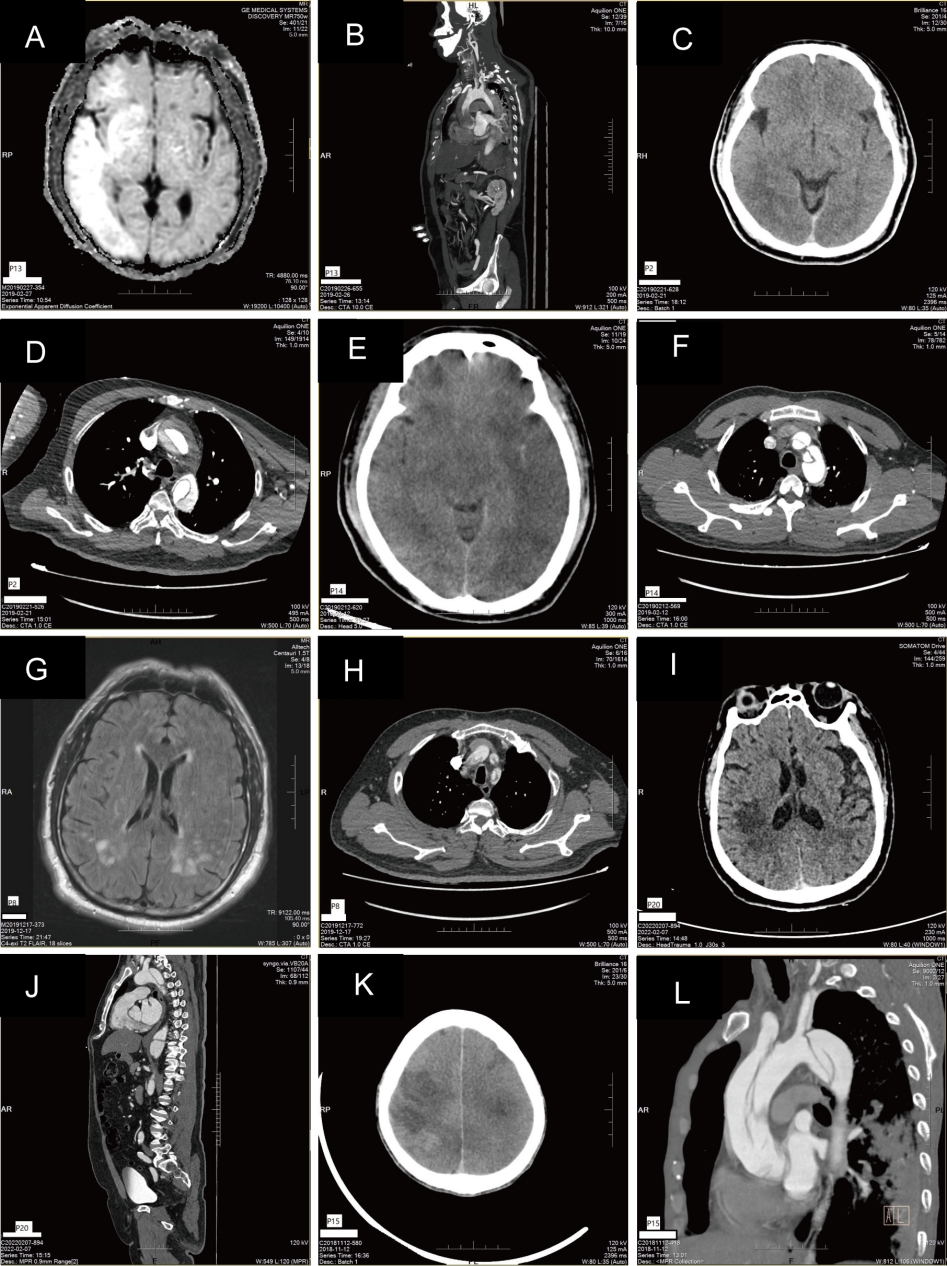
**Imaging of aortic dissection and cerebral infarction**. (A) MRI diffusion weighted imaging (DWI) showed a large area of hyperintensity in the right temporal-parietal lobe and occipital lobe, and scattered hyperintensity in the other bilateral cerebral hemispheres and cerebellar hemispheres, suggesting acute cerebral infarction. (B) Aortic dissection type A with three involved vessels in the arch. (C) CT showed hypodense lesions in the right parietal lobe and posterior horn of the lateral ventricle. (D) Aortic dissection type A with three involved vessels in the arch. (E) CT left cerebral hemisphere low density focal cerebral infarction? Abnormal signal focus of the right frontal lobe hemorrhagic infarction? (F) Aortic dissection type A. (G) DWI showing bilateral parietal occipital lobe and pons hyperintensity. (H) Aortic dissection type A, involving the brachiocephalic trunk, bilateral common carotid arteries, and bilateral subclavian arteries. (I) Right frontal-parietal hypodense lesion on CT. (J) Aortic dissection type B. (K) CT bilateral frontal lobe, temporal lobe, parietal lobe, and left basal ganglia multiple low-density foci acute cerebral infarction? (L) Aortic dissection type A brachiocephalic trunk, right subclavian artery, left internal carotid artery, and left subclavian artery involvement. (A and B, C and D, E and F, G and H, I and J, and K and L were from different patients). MRI, magnetic resonance imaging; DWI, diffusion weighted imaging; CT, computed tomography.

Of the 25 patients with cerebral infarction as the primary presentation of acute AD, 17 (68%) showed cerebral infarction changes in head CT or MRI. No obvious signs of cerebral infarction were found in the remaining 8 patients, but 7 of these involved the brachiocephalic trunk and one involved the common carotid artery. Patient information is shown in Table 1, with a typical MRI shown in Fig. 1. Seventeen of the 25 patients (68%) had hypertension (high blood pressure, HBP), while 4 patients had no prior medical history. Unfortunately, 18 patients (72%) were diagnosed as having a stroke and therefore a diagnosis of AD was missed. Of the 18 patients with missed diagnoses, 12 subsequently died (67%), compared to 5 of the 7 patients who were diagnosed with AD (71%). Hence, there was no statistical difference in mortality between the patients with cerebral infarction who were or were not diagnosed as having AD (chi-square test, *p *> 0.9999).

**Table 1.** **Clinical characteristics, outcome of each patient**.

Case	Sex	Age	Symptoms	Missed diagnosis of AD	Dissection type	Involved cerebrovascular	GERAADA score (%)	Prognosis	Death reasons
1	F	69	Headache for 5 days	yes	Type A	BCA, LCCA, LSCA	19.6	alive	
2	M	54	Left limb weakness 7 hours, abdominal pain for 5 hours	yes	Type A	RCCA, RSCA	53	died	aortic rupture, cardiac tamponade
4	M	53	Dizziness and chest tightness for 2 days	yes	Type A	BCA, LCCA, LSCA	24.3	alive	
5	F	40	Disorder of consciousness for 2 hours	yes	Type A	BCA, LCCA, LSCA	17.7	died	broad cerebral infarction
6	M	33	Transient syncope and chest pain for 4 hours	yes	Type A	BCA	11.4	alive	
10	F	60	Prominent left limb weakness for 11 hours	yes	Type A	BCA, RCCA	78.1	died	aortic rupture, cardiac tamponade
12	M	56	Left lower extremity numbness and chest and back pain for 3 hours	yes	Type A	BCA, LCCA, RSCA, LSCA	79.8	died	cardiac tamponade, myocardial infarction
13	M	50	Disorder of consciousness for 20 hours	yes	Type A	BCA, LCCA, LSCA	81.2	died	broad cerebral infarction
14	M	45	Coma for 2 days	yes	Type A	LCCA, LSCA	79.8	died	broad cerebral infarction, myocardial infarction
15	M	50	Coma for 9 hours	yes	Type A	BCA, LCCA, LSCA	43.1	died	broad cerebral infarction
16	M	60	Disturbance of consciousness for 10 days	yes	Type A	BCA, LCCA, LSCA	74.6	died	aortic rupture, hypotensive shock
17	M	72	Disturbance of consciousness for 6 days	yes	Type A	BCA, LSCA	92.8	died	aortic rupture, cardiac tamponade
22	F	58	Right limb weakness for 8 days	yes	Type A	No cerebrovascular teared	35.4	alive	
23	F	56	Sudden left limb weakness with chest pain for 2 hours	yes	Type A	BCA, LCCA, LSCA	34.5	alive	
24	F	64	Fatigue with chest and abdomen pain for 1 day	yes	Type A	BCA, LCCA, RCCA	92.1	died	cardiac tamponade, broad cerebral infarction
7	F	84	Left limb weakness for 1 month and chest pain for 10 days	yes	Type B	No cerebrovascular teared	26	died	broad cerebral infarction
20	M	58	Slurred speech and physical weakness for 3 days	yes	Type B	LCCA, RSCA	34.1	alive	
21	F	63	Sudden disturbance of consciousness for 2 hours	yes	Type B	No cerebrovascular teared	81.4	died	broad cerebral infarction
8	M	56	Post-traumatic chest pain for 16 days, Consciousness disturbance 9 hours	no	Type A	BCA, LCCA, LSCA	18	alive	
9	F	67	Head, neck and chest pain for 7 hours	no	Type A	BCA, LCCA	56	died	right ventricular myocardial infarction
11	M	75	Consciousness disturbance with chest pain for 6 hours	no	Type A	Not applicable	84.7	died	aortic rupture, cardiac tamponade
18	M	80	Transient syncope and Chest pain for 4 hours	no	Type A	Not applicable	33.2	died	aortic rupture, hypotensive shock
19	F	69	Transient syncope and Chest pain for 7 hours	no	Type A	No cerebrovascular teared	20.7	died	broad cerebral infarction
25	M	65	Disturbance of consciousness for 10 hours	no	Type A	BCA, LCCA	90.7	died	aortic rupture, cardiac tamponade
3	M	41	Transient syncope, then chest pain for 2 days	no	Type A	BCA, LCCA, LSCA	29.7	alive	

AD, aortic dissection; GERAADA score, the German Registry of Acute Aortic Dissection Type A score; F, female; M, male; BCA, brachiocephalic trunk; LCCA, left common carotid artery; LSCA, left subclavian artery; RCCA, right common carotid artery; RSCA, right subclavian artery.

The involved cerebrovascular lesion was reported as the involved brachiocephalic trunk (BCA, divided into right common carotid artery [RCCA] and right subclavian artery [RSCA]), the left common carotid artery (LCCA), and the left subclavian artery (LSCA).

Demographic information and laboratory test results were compared between AD patients with cerebral infarction as the first presentation (n = 25) and control patients (n = 933). As shown in Table 2, there were no significant differences in age and sex between the two groups. Patients presenting with cerebral infarction had a higher incidence of type A AD than the controls (*p* = 0.0002). Systolic blood pressure (SBP) and diastolic blood pressure (DBP) in patients with cerebral infarction as the first presentation were both lower than controls (*p* = 0.0041 and *p* = 0.0001, respectively), while their mortality rate was higher (*p *< 0.0001). Furthermore, patients with cerebral infarction had significantly higher levels of blood sugar, lactate dehydrogenase, myoglobin, creatine kinase-MB, prothrombin time, international normalized ratio (INR), fibrinogen degradation products (FDP) and D-dimer than control patients (all *p *< 0.05). However, no statistically significant differences were observed between the two groups for the other routine laboratory blood tests, including liver function (alanine aminotransferase and aspartate aminotransferase), kidney function (serum creatinine, blood urea nitrogen and uric acid), coagulation (fibrinogen, activated partial prothrombin time, and thrombin time), creatine kinase, total bilirubin, triglycerides, total cholesterol, high-density lipoprotein and C-reactive protein.

**Table 2.** **Comparisons between AD patients with cerebral infarction as the first presentation and AD patients with other symptoms at the first presentation**.

	Cerebral infarction as the first presentation (n = 25)	Other symptoms as the first presentation (n = 933)	*p*
Age	58 (51.5, 68) *	56 (48, 67) *	0.3760
Sex (male)	15	694	0.1102
Type A aortic dissection	22	471	0.0002
Type B aortic dissection	3	462	
P (/min)	81 (68, 88) *, (n = 25)	80 (69, 92) *, (n = 896)	0.6556
R (/min)	20 (17.5, 22) *, (n = 25)	20 (8, 22) *, (n = 896)	0.4338
SBP (mmHg)	133.0 (103, 148.5) *, (n = 25)	145.0 (123, 169) *, (n = 892)	0.0041
DBP (mmHg)	69.00 (56, 79.5) *, (n = 25)	82.00 (69, 95.79) *, (n = 892)	0.0001
Died	17	95	<0.0001
Alived	8	838	
Laboratory results (available data)			
Blood sugar (mmol/L)	7.89 (6.625, 10.5) *, (n = 22)	6.99 (6.17, 8.42) *, (n = 735)	0.0142
Lactate dehydrogenase (U/L)	252 (198, 308) *, (n = 23)	224 (183, 272) *, (n = 749)	0.0426
Myoglobin (μg/L)	107.5 (37.3, 280.8) *, (n = 23)	48.20 (27.65, 96.2) *, (n = 749)	0.0109
Creatine kinase-MB (U/L)	16.30 (14.5, 28.7) *, (n = 23)	13.70 (9.8, 18.8) *, (n = 749)	0.0109
Prothrombin time (s)	13.95 (12.3, 15.5) *, (n = 24)	13.00 (11.9, 14.2) *, (n = 767)	0.0332
International normalized ratio (INR)	1.145 (1.053, 1.248) *, (n = 24)	1.070 (0.99, 1.15) *, (n = 768)	0.0224
Fibrinogen degradation products (FDP) (mg/L)	20.85 (10.6, 45.2) *, (n = 24)	11.50 (5.0, 28.55) *, (n = 769)	0.0335
D-dimer (mg/L)	1.99 (0.91, 4.668) *, (n = 24)	1.44 (0.57, 2.73) *, (n = 775)	0.1022

* Mann-Whitney U-test was used as the data were not normally distributed. The results are shown as median (quartile) and [*M* (*Q*1, *Q*3)]. AD, aortic dissection; P, pulse; R, respiration; HBP, high blood pressure; SBP, systolic blood pressure; DBP, diastolic blood pressure; FDP, fibrinogen degradation products.

## 4. Discussion

AD is mainly caused by a tear in the aorta. However, the lesion can also lead to tearing of large arteries originating from the aorta, resulting in dysfunction of the organs to which the blood is normally directed. Blood is mainly supplied to the brain by the common carotid artery and the aorta from the subclavian artery. Therefore, AD involving the brachiocephalic trunk, the common carotid artery, or the subclavian artery may restrict blood supply to the brain and cause cerebral malperfusion [14]. In the present study, just 2.6% of AD patients (4.67% of type A and 0.65% of type B) presented with cerebral infarction as the first clinical manifestation. To date, the incidence of cerebral infarction as the first presentation of AD has been reported in only a small number of cases [15, 16]. Much of the literature has been concerned with cerebral infarction in the perioperative period of AD [11, 17]. Of 775 patients who presented with acute type A AD, 80 (10%) had cerebral malperfusion [9]. In the Nordic Consortium study of acute type A AD, stroke occurred in 15.7% of patients (177/1128) [18]. Thus, although we found the incidence of cerebral infarction as the first clinical manifestation of AD was not insignificant at 2.6%, this symptom is several-fold more common in the perioperative period.

Our study cohort of 25 AD patients with cerebral infarction contained 22 cases with type A dissection and 3 with type B dissection. Type A dissection is more common in cases with cerebral infarction, probably due to anatomical reasons. Brain tissue is supplied by the internal carotid artery branching from the common carotid artery, as well as by the vertebral artery branching from the subclavian artery. Although type A dissection can also be divided into DeBakey types I and II, most cases are type I. Type A dissection often involves the ascending aorta, aortic arch and descending aorta, with the aortic arch giving off the brachiocephalic trunk, left common cervical artery, and left subclavian artery from right to left. The brachiocephalic trunk is divided into the right cervical and right subclavian arteries. Any dissection tear that accumulates in the vessels above the aortic arch will result in brain tissue ischemia and may result in ischemic cerebral infarction. Type B dissection is defined as being limited to the descending aorta (no accumulation in the ascending aorta or aortic arch), such that intimal rupture is located at the distal end of the left subclavian artery. Brain tissue ischemia followed by ischemic cerebral infarction only occurs when dissection leads to systemic under-perfusion or arterial arch stenosis. Therefore, type A dissection is predisposed anatomically to cause cerebral infarction.

The incidence of cerebral infarction as the first presentation of AD is extremely low, but unfortunately the rate of missed diagnosis of AD in such cases is relatively high [4, 19]. In the present study, head CT or MRI showed cerebral infarction changes in 17 of 25 patients. Furthermore, 18 patients were initially diagnosed with stroke, and a diagnosis of AD was missed. The relatively high rate of missed diagnosis for AD could be because many of the cerebral infarction patients had disturbed consciousness and were unable to express themselves or to speak. In addition, some patients did not have chest or back pain. For patients with suspected cerebral infarction, the time window for thrombolysis is very limited [20], and basic chest examination or other laboratory tests were not performed. Although cerebral infarction is a quite common disease, cerebral infarction caused by AD is rare. Many patients are first referred to neurologists, who tend to pay considerable attention to cerebral infarction or cerebral hemorrhage and to ignore AD, which is a relatively rare cause of cerebral infarction. Moreover, neurologists usually only perform a head CT and/or a plain CT scan of the chest, which cannot easily detect a dissection. Although AD cases are not uncommon in large hospitals, they are relatively rare in primary hospitals. Many primary hospitals are unable to perform CT angiography, and therefore it is not easy to make a definite diagnosis of AD. Until recently, there have been no serological markers for the early detection of AD and cerebral infarction [1]. The most commonly used serological marker for AD is D-dimer, however, this marker is also elevated in patients with cerebral infarction [21]. In clinical practice, bilateral SBP differentials >20 mmHg are associated with non-traumatic type A AD [22]. This may be useful for the early identification of AD. However, it is important to be aware that cerebral infarction may be a clinical manifestation of dissection, otherwise blood pressure will usually only be measured in one upper limb.

Unfortunately, AD patients with cerebral infarction as the first presentation had a high death rate. These patients had lower SBP and DBP, and higher levels of blood sugar, lactate dehydrogenase, myoglobin, creatine kinase-MB, prothrombin time, INR and FDP than control AD patients. This finding indicates that cerebral infarction patients had multiple organ dysfunction and a worse general condition. In agreement with this observation, it has been reported that stroke in acute type A AD patients is associated with increased early- and mid-term mortality [18]. Other authors have reported that initial SBP and DBP were lower in AD patients with central nervous system symptoms [14]. In the present study, patients 11 and 18 received alteplase (rt-PA) initiation or anticoagulation treatment, with patient 11 dying from aortic rupture and hypotensive shock. The cerebral infarction thrombolysis time window is generally not more than 6 hours. However, in our study the time from illness to hospital admission was more than 6 hours for most patients. Some patients therefore presented with altered consciousness or headache rather than classic symptoms such as hemiplegia and crooked mouth. This meant that most patients did not receive thrombolytic therapy, but all patients received the routine anticoagulant and antiplatelet aggregation therapy. However, our results showed that AD patients with cerebral infarction as the first presentation had a high mortality rate, regardless of whether the diagnosis of AD had been missed. This suggests the patient’s risk of death was mostly associated with the extent of dissection involvement. Once the brachiocephalic trunk, common carotid artery or subclavian artery are torn, this results in a serious lack of blood supply to the brain tissue, and thus a poor outcome is unavoidable.

Our study has several limitations. AD patients generally have bilateral blood pressure differences >20 mmHg [22], and D-dimer is also significantly increased in the acute phase [23]. Bilateral limb blood pressure is usually measured in order to reduce misdiagnosis and missed diagnosis of AD. However, bilateral blood pressure was not recorded here due to the retrospective nature of the study. Furthermore, D-dimer levels were significantly increased in the patients, and the physician did not initially consider aortic dissection. The cerebral infarction was sometimes first diagnosed in another hospital, and the D-dimer test was not performed at that time. Because the laboratory test results was missing for many patients, regression analysis could not be performed to determine the most relevant parameter for cerebral infarction in AD patients. Finally, we were unable to obtain more differentiated biomarkers because the number of AD patients with cerebral infarction at first presentation was relatively small.

## 5. Conclusions 

AD presenting initially as cerebral infarction is a rare condition, with such patients having a high risk of death. However, failure to initially diagnose AD in these patients did not further increase mortality.

## Data Availability

The datasets used and/or analyzed during the current study are available from the corresponding author on reasonable request.
